# The transcription factor XBP-1 in neurodegenerative diseases

**DOI:** 10.1186/1750-1326-8-S1-O24

**Published:** 2013-09-13

**Authors:** Frédéric Checler, Eric Duplan, Cristine Alves da Costa

**Affiliations:** 1 IPMC, UMR7275 CNRS/UNSA, Valbonne, France

## 

Parkinson’s disease (PD) is a neurodegenerative disorder that is either associated with an autosomal dominant or recessive mode of inheritance. The latter forms are mostly linked to mutations in the genes of *parkin*, *Pink-1* and *DJ-1.* Several lines of evidence indicate that *DJ-1* could act as an antioxidant while parkin has been characterized as an ubiquitin-ligase. Parkin was reported to interact physically with *DJ-1* monomers in oxidative stress conditions but did not promote its ubiquitin-linked proteasomal degradation. This led us to examine whether parkin could control *DJ-1* via its function as transcription factor that we documented recently [1]. We have shown [2] that parkin controls *DJ-1* by a mechanism independent of its ubiquitin-ligase activity and that this regulation is abolished by PD-related pathogenic mutations. Thus, parkin increases *DJ-1* promoter *trans*-activation, mRNA levels and protein expression via a transcriptional cascade involving p53 repression and subsequent activation of ER-stress induced X-box-binding protein-1S (XBP-1S). Then, XBP-1S physically interacts with *DJ-1* promoter, thereby raising *DJ-1* mRNA and protein levels. Overall, our study unravels a functional dialogue by which parkin and *DJ-1* could control ER-stress response in physiopathological conditions. We will discuss the potential involvement of XBP-1 in other neurodegenerative diseases.
